# Crosstalk Between GABAergic Neurotransmission and Inflammatory Cascades in the Post-ischemic Brain: Relevance for Stroke Recovery

**DOI:** 10.3389/fncel.2022.807911

**Published:** 2022-03-23

**Authors:** Georgios Michalettos, Karsten Ruscher

**Affiliations:** ^1^Laboratory for Experimental Brain Research, Division of Neurosurgery, Department of Clinical Sciences, Wallenberg Neuroscience Center, Lund University, Lund, Sweden; ^2^LUBIN Lab—Lunds Laboratorium för Neurokirurgisk Hjärnskadeforskning, Division of Neurosurgery, Department of Clinical Sciences, Lund University, Lund, Sweden

**Keywords:** stroke recovery, inflammation, GABA, immune cell, neurotransmission, chemokine, glutamate decarboxylate

## Abstract

Adaptive plasticity processes are required involving neurons as well as non-neuronal cells to recover lost brain functions after an ischemic stroke. Recent studies show that gamma-Aminobutyric acid (GABA) has profound effects on glial and immune cell functions in addition to its inhibitory actions on neuronal circuits in the post-ischemic brain. Here, we provide an overview of how GABAergic neurotransmission changes during the first weeks after stroke and how GABA affects functions of astroglial and microglial cells as well as peripheral immune cell populations accumulating in the ischemic territory and brain regions remote to the lesion. Moreover, we will summarize recent studies providing data on the immunomodulatory actions of GABA of relevance for stroke recovery. Interestingly, the activation of GABA receptors on immune cells exerts a downregulation of detrimental anti-inflammatory cascades. Conversely, we will discuss studies addressing how specific inflammatory cascades affect GABAergic neurotransmission on the level of GABA receptor composition, GABA synthesis, and release. In particular, the chemokines CXCR4 and CX3CR1 pathways have been demonstrated to modulate receptor composition and synthesis. Together, the actual view on the interactions between GABAergic neurotransmission and inflammatory cascades points towards a specific crosstalk in the post-ischemic brain. Similar to what has been shown in experimental models, specific therapeutic modulation of GABAergic neurotransmission and inflammatory pathways may synergistically promote neuronal plasticity to enhance stroke recovery.

## Introduction

Stroke is among the most common and important causes of disability and death worldwide. Annually, approximately 12 million people suffer a stroke worldwide; thereof 6 million patients do not survive the insult. Approximately 5 million stroke victims acquire life-long disabilities and will need support for daily living by next of kin or at institutions (Feigin et al., 2021). The societal cost associated with stroke is huge, amounting to 60 billion euros in the EU in 2019 (Luengo-Fernandez et al., 2020), alarming figures we expect to grow with an increasing aging population and health care costs. Stroke causes loss of neurons and of neurological function due to cell loss predominantly in the affected neuronal tracts and circuits. In addition, neurological deficits are also due to dysfunction of remaining neurons in the vicinity to or in areas remote from the infarct connected through brain-wide neural networks (Carter et al., 2010). The affected neuronal networks, therefore, are considered as neuronal substrates for recovery-promoting therapies modulating mechanisms of brain plasticity, i.e., the innate ability of the brain to remodel neural network connections.

Brain plasticity comprises the ability of the brain to reorganize its cellular structures and its function in response to intrinsic and extrinsic stimuli (Wieloch and Nikolich, 2006; Cramer et al., 2011). Brain plasticity can be stimulated in stroke patients by multimodal rehabilitation (Bunketorp-Kall et al., 2017). For instance, various advanced training paradigms, assisted by virtual reality, computer gaming, are thought to stimulate brain plasticity, and have shown promise in supporting rehabilitation (Hatem et al., 2016). In the experimental setting, this is accomplished by an enriched environment (EE), comprising large cages with toys, tubes, ladders, and larger groups of animals with the opportunity for more complex social interaction that activates various neural networks of the brain. Furthermore, transcranial magnetic stimulation (TMS) or direct current stimulation (tDCS) enhances recovery even when treatment is instituted months after the stroke. However, pharmacological interventions are still limited to support rehabilitation after stroke.

Appropriate neuronal function depends on interconnected and well-organized circuits of inhibitory interneurons and excitatory projection of cortical pyramidal neurons. Any changes in the synaptic stability and organization of inhibitory neurons may impair the regulation of excitatory circuits. GABAergic neurotransmission, therefore, is a key element regulating the excitation/inhibition balance, and brain connectivity.

### Background—Elements of GABAergic Neurotransmission

γ-aminobutyric acid (GABA) is the main inhibitory neurotransmitter in the adult brain. Together with the excitatory neurotransmitter glutamate, GABA neurotransmission regulates the inhibitory-excitatory balance necessary for adequate brain function. In the adult brain, GABA is responsible for the hyperpolarization of the cell, preventing the conveyance of neuronal action potentials. The main functions of GABA in mediating inhibitory neuronal activity have been extensively studied. In addition, research is focused on unraveling new roles of the amino acid in non-neuronal cell functions and pathologies. There are two main types of GABA receptors ionotropic GABA_A_(GABA_A_Rs) receptors and metabotropic GABA_B_ receptors (GABA_B_Rs; Wu and Sun, 2015).

### Glutamic Acid Decarboxylase Isoforms

Synthesis of GABA in neurons is catalyzed by two isoforms of the glutamicacid decarboxylase, GAD65, and GAD67 (Pinal and Tobin, 1998). These key enzymes convert glutamate to GABA. Different functions of each isoform appeared mainly due to studies from knock-out mice (Asada et al., 1997; Condie et al., 1997; Kash et al., 1997; Tian et al., 1999). GAD67 has been found to be constitutively active and provide the majority of the cytosolic GABA, while GAD65 is mainly responsible for synaptic GABA production released from synaptic vesicles during neurotransmission (Pinal and Tobin, 1998; Battaglioli et al., 2003). Together, both isoforms exhibit different cellular distribution and structural properties (Dupuy and Houser, 1996; Jin et al., 2003). Their function strongly depends on post-translational modifications including phosphorylation, palmitoylation, and cleavage (Lee et al., 2019).

While GAD67 is a hydrophilic cytoplasmic protein, GAD65 undergoes post-translational hydrophobic modifications, which enable it to be anchored on the membrane compartments of the synaptic vesicles (Kanaani et al., 1999, 2002). Further studies have confirmed that the two isoforms can interact with each other, creating heterodimers in the membrane of the synaptic vesicles (Kanaani et al., 2004, 2010). This indicates that GAD67 is not only involved in maintaining metabolic levels of cellular GABA but may also contribute to the rapid vesicular accumulation of GABA in the presynaptic terminal for covering the incremental needs of synaptic neurotransmission by vesicular release into the synaptic cleft (Kanaani et al., 2010).

### GABA_A_ Receptors

GABA_A_ receptors belong to the family of Cys-loop ligand-gated ion channels and are responsible for mediating most of the fast inhibitory neurotransmission in the central nervous system (CNS). When GABA binds to these receptors at post-synaptic sites, the ion channel opens, enabling the influx of chloride (Cl^−^) ions into the cell along a concentration gradient resulting in a change in the membrane potential. Together with other factors such as the maturation status of cells, either de- or hyperpolarization of the post-synaptic mature neuron occurs (Fritschy and Panzanelli, 2014). GABA_A_ receptors are pentameric channels which are composed of a variety of subunits. Nineteen genes have been identified encoding GABA_A_ receptor subunits (α1–α6, β1–β3, γ1–γ3, δ, ε, π, θ, ρ1–ρ3) in mammals, demonstrating that there is high heterogeneity assembling the receptor (Barnard et al., 1998; Bonnert et al., 1999). Experimental evidence suggests that the GABA_A_ receptor assembled of different subunit compositions provide variable functions and pharmacological properties (Gingrich et al., 1995; Browne et al., 2001; Dixon et al., 2014). The second type of GABA_A_ receptor previously described as the GABA_C_ receptor, is a homopentameric ion channel solely comprised of ρ1-ρ3 subunits with distinct distribution in the CNS (Wegelius et al., 1998).

Depending on their cellular localization, GABA_A_ receptors have been categorized into synaptic and extra-synaptic, mediating synaptic (phasic), and extra-synaptic (tonic) inhibition, respectively (Farrant and Nusser, 2005; Glykys and Mody, 2007; Brickley and Mody, 2012). The most common stoichiometry, accounting for the majority of the overall distribution of GABA_A_ receptors in the brain, is the heteropentameric form consisting of two α-subunits, two β- and one γ-subunit, with the α1α1β2β2γ2 combination (Sieghart and Sperk, 2002; Goetz et al., 2007). Initially, segregation was made between synaptic and extrasynaptic receptors with synaptic (phasic) GABA_A_ receptors being composed primarily of α1–3, β1–3, and γ1–3 subunits and extrasynaptic GABA_A_ receptors consisting of α4, α5, α6, or δ-subunits. However, electrophysiological studies combined with pharmacological application of GABA_A_ receptor positive allosteric modulators have revealed that subunits, previously defined as “synaptic” are also found in somatic membranes of neurons (Lindquist and Birnir, 2006; Stojanovic et al., 2016). In specific, zolpidem, a positive allosteric compound of α1-, α2-, α3-, and γ2- containing GABA_A_ receptors, activated such receptors in granule neurons of the dentate gyrus, however, to a lesser extent compared to compounds that target α4-, α6-, and δ- containing receptors (Lindquist and Birnir, 2006). These results indicate that a higher receptor subunit diversity of extrasynaptic receptors.

Depending on the neuronal cell type, GABA_A_ receptors can be localized in different subcellular regions such as in somata, dendrites, synapses, and in the non-synaptic membrane (Somogyi et al., 1989). Furthermore, the δ-subunit is assembled in the place of the γ2 subunit and is typically associated with the α4 and α6-subunit isoforms (Clarkson, 2012; Fritschy and Panzanelli, 2014). Several studies have revealed that the β2, β3, γ2 variants are the most abundant isoforms participating in the assembly of the majority of GABA_A_ receptors subtypes, with α4βδ complexes being mostly located in the cortex, hippocampus, and thalamus and α6βδ complexes being located in the cerebellum (Fritschy and Panzanelli, 2014; Nguyen and Nicoll, 2018).

Similar distribution patterns of GABA_A_ receptor subunits have been found in rodents and immunohistochemical and *in situ* hybridization studies on post-mortem human tissue (Waldvogel et al., 2010). Most of the αsubunits exhibit laminar expression in the cerebral cortex similar to that of rodents (Akbarian et al., 1995; Lewohl et al., 1997; Waldvogel et al., 2010; Stojanovic et al., 2016). In addition, structures such as the hippocampus, the basal ganglia, and the thalamus in the human brain exhibit a high degree of similarity to rodents regarding the expression profile of GABA_A_ receptor subunit subtypes and their subcellular localization (Houser et al., 1988; Waldvogel et al., 1999; Loup et al., 2000; Popken et al., 2002; Stojanovic et al., 2016). However, it should be noted that differences have been observed, mostly on the expression level of individual subunit subtypes (Waldvogel et al., 2010). Furthermore, due to the difficulty of conducting relevant experiments in humans, there is limited data on the dynamics of receptor composition in the human brain, an aspect taken into consideration when translating preclinical results to the human brain.

### Topological Dynamics of GABA_A_ Receptor Subunits

The localization of GABA_A_ receptors is a dynamic process, which involves the trafficking of the ion-channel receptors along the surface of the neurons (Choii and Ko, 2015; Lorenz-Guertin and Jacob, 2018). When focusing on synaptic inhibition, GABA_A_ receptors, either through lateral diffusion or exocytosis, accumulate in the membrane of the post-synaptic neuron. Synaptic localization of the receptor is determined by its interaction with a “local” anchoring complex, consisting of gephyrin molecules (Craig et al., 1996; Studler et al., 2005; Goetz et al., 2007).

Several studies have focused on the importance of the γ2-subunit maintaining the ability to cluster on the inhibitory synapses (Kittler and Moss, 2003; Kittler et al., 2004). Lack of the γ2-subunit and replacement by δ-containing GABA_A_ receptors, along with their respective α4, α5, and α6 variants, prevents localization at the synaptic cleft and such GABA_A_ receptors are found almost exclusively extrasynaptically, mediating tonic inhibition through ambient GABA levels (Nusser and Mody, 2002; Stell et al., 2003; Zheleznova et al., 2009). However, this model seems not rigid, since GABA_A_ receptors containing ofα1β2γ2 or α3β3γ2 subunits can also be found in extrasynaptic membranes, although less frequently, thus determining a dynamic system regarding the localization of GABA_A_ receptors (Nusser et al., 1998; Mortensen et al., 2012).

The above-mentioned distribution pattern of extrasynaptic receptors is found in cerebellar granular cells, the dentate gyrus, the thalamus, in the granular cortical layers as well as in the hippocampus (Nusser and Mody, 2002; Sieghart and Sperk, 2002; Zheleznova et al., 2009). The α1-α3 subunits exhibit unique distribution patterns and partial overlap, with the α1 subunit being the most prevalent throughout the adult mouse forebrain (Sieghart and Sperk, 2002; Fritschy and Panzanelli, 2014).

### Spatiotemporal and Age-Related Alterations in GABA_A_ Receptor Subunits Expression

The GABAergic neurotransmitter system is highly modular and dynamic. Variables such as age, sex, environmental factors, dietary habits, circadian cycle, contribute to the complexity of successfully translating preclinical findings into precise personalized clinical care. For example, spectroscopy studies on humans regarding the response of GABA to motor learning revealed a decrease of the inhibitory neurotransmitters in the motor cortex (M1) in intervals of neuronal plasticity changes (Sampaio-Baptista et al., 2015; Kolasinski et al., 2019). Furthermore, the progression of the ovarian cycle alters the number of δ-containing GABA_A_Rs in the hippocampus, ultimately affecting the electrophysiological properties of the local circuitry (Barth et al., 2014). In parallel, GABA_A_R subunit expression is modulated by estrogens, however, in-depth studies are warranted if these changes depend on the brain region (Herbison and Fénelon, 1995). Subunit expression does not differ between males and females in the temporal cortex, although differences in other brain regions cannot be excluded (Pandya et al., 2019). Furthermore, alcohol dependence studies focused on post-mortem human brains have revealed spatial alterations in the expression of certain GABA_A_ subunits, indicating possible changes in receptor composition that could be indicative of the behavioral pathology in dependence (Jin et al., 2012; Bhandage et al., 2014).

Interestingly, subunits such as the ε and ρ2 subtypes which are less frequently studied were not only confirmed on transcript level in the human brain but were also demonstrated to undergo regulation (Bhandage et al., 2014). Nevertheless, the extent to which functional subunits are integrated into receptors and are modulated by the same post-translational modifications observed in rodents remains to be elucidated in humans.

In the context of aging, evidence shows that the GABAergic system might be affected in older age, unable to exert its fine-tuned inhibitory efficacy on the neural circuits of the brain, ultimately affecting plasticity and adaptiveness to brain injury. In specific, electrophysiological studies conducted on transgenic APPSwe mice, a rodent model of Alzheimer’s disease (AD), revealed that GABA-induced currents in the dentate gyrus (DG) of the hippocampus are not altered in this area by age alone but in combination with the presence of Alzheimer’s disease pathology (Hammoud et al., 2021). In accordance with this observation, electrophysiological recordings on human temporal cortices with AD demonstrated impaired GABAergic signaling (Limon et al., 2012). This effect was attributed to possible alterations in the composition of GABA_A_ receptors, overall affecting the brain’s response to GABA (Limon et al., 2012). On the other hand, quantitative studies on human cortical samples revealed that the expression of most of the elements comprising the GABAergic system remains robust throughout age with the exception of certain subunits and GAT-1 (Pandya et al., 2019). Additionally, alterations in subunit expression differ throughout aging between males and females (Ethiraj et al., 2021).

### GABA_B_ Receptors

GABA_B_ receptors belong to the family of G protein-coupled receptors (GPCR). They are responsible for the later and slower component of inhibitory transmission and are found both on the pre- and post-synaptic membrane (Huang et al., 1995; Sakaba and Neher, 2003; Ulrich and Bettler, 2007). GABA_B_ receptors are comprised of two subunits, R1 and R2. Their regulation varies on the transcriptional and post-translational levels dependent on the physiological or pathological condition (Benke et al., 1999; Billinton et al., 2000; Terunuma et al., 2010; Kantamneni et al., 2014). The majority of GABA_B_ receptors inhibit isoforms of the adenyl cyclase (AC) through the G_αi/o_ subunits (Wojcik and Neff, 1984; Xu and Wojcik, 1986; Terunuma, 2018). In parallel, different isoforms of AC have been demonstrated to undergo stimulation as a result of ligand binding due to activation from G_βγ_ subunits of GPCR receptors (Tang and Gilman, 1991; Terunuma, 2018), indicating a multi-functional role of these receptors in modulating intracellular signaling pathways and neuronal activity.

Activation of GABA_B_ receptors is also coupled to K^+^ and/or Ca^2+^ channels *via* G-protein mediated pathways (Gähwiler and Brown, 1985; Huang et al., 1995; Misgeld et al., 1995; Sodickson and Bean, 1996; Filippov et al., 2000). Both channels are either activated or inhibited by β and γ subunits of the G protein. Upon opening of G protein-gated inward rectifying potassium (GIRK) channels, membrane potential changes, reducing the excitability of neurons in their resting phase. In addition, GABA_B_ receptors inhibit voltage-sensitive calcium (Ca^2+^) channels (VSCC) controlling the rate of neuronal firing as well as neuronal processes dependent on the dynamics of intracellular calcium (Mintz and Bean, 1993; Pfrieger et al., 1994; Lambert and Wilson, 1996; Limon et al., 2012; Bhandage et al., 2014; Hammoud et al., 2021). As such, both channels have been demonstrated to be determinant elements in defining synaptic transmission and plasticity in neuronal networks under physiological conditions as well as brain pathologies (Chung et al., 2009; Frank, 2014; Marron Fernandez de Velasco et al., 2015; Sánchez-Rodríguez et al., 2017; Nanou and Catterall, 2018).

In this review, we will focus on the relevance of the aforementioned elements of the GABAergic system in the context of brain injury, specifically ischemia and stroke, and their possible interactions with the inflammatory and immune response found in the post-ischemic brain during the recovery phase of stroke. We will focus on the involvement of GABA_A_ receptors as mediators of neuronal activity and synaptic plasticity and the perspective of pharmacological intervention, contributing to neuroprotection and the recruitment of cellular repair mechanisms necessary to facilitate stroke outcome.

### GABAergic Neurotransmission After Stroke

An initial increase in the release of GABA is found in the ischemic brain following global ischemia (Globus et al., 1991) and focal ischemia induced by permanent occlusion of the middle cerebral artery (Ruan et al., 2017). Interestingly, to counteract the excitotoxic insult various studies demonstrate that an enhanced GABAergic tonus i.e., by administration of GABAergic agents prior to ischemia or shortly after the ischemic episode provides neuro protection in preclinical stroke models (Corbett et al., 2008). In addition, the identification of molecules and cascades that enhance GABAergic neurotransmission during the acute phase after stroke has been a promising field defining neuroprotective compounds. For example, peptide hormones, such as oxytocin and insulin, have been characterized for their *in vitro* neuroprotective role through mechanisms of GABA_A_ receptor subunit upregulation and increased post-ischemic cell-surface receptor stability, respectively (Mielke and Wang, 2005; Kaneko et al., 2016). In addition, erythropoietin, a glycoprotein whose application has been extensively investigated in stroke for its protective effect, has been demonstrated to enhance GABAergic activity (Ruscher et al., 2002; Gonzalez et al., 2007; Juenemann et al., 2020; Roseti et al., 2020). While levels of GABA increased, the immediate intrinsic reaction of insulted cells appears to downregulate and internalize cell-surface GABA_A_ receptors after the insult (Kittler et al., 2005; Mielke and Wang, 2005; Mele et al., 2014; Costa et al., 2016). Decreased density of plasma-membrane receptors has been associated with truncation of anchoring structures as well as post-translation modifications on regulatory residues of receptor subunits that promote clathrin-dependent endocytosis (Kittler et al., 2005; Mielke and Wang, 2005; Mele et al., 2014; Costa et al., 2016). Despite promising preclinical results, early administration of benzodiazepines did not favor outcomes in patients and even increased post-stroke mortality at 90 days (Lodder et al., 2006; Colin et al., 2019). The discussion of unsuccessful translation is beyond the scope of this review and will need to involve all aspects of translation of preclinical data into clinical development and practice.

Modulation of processes that contribute to recovery beyond the time window of neuroprotection represents a paradigm shift aiming at enhancing brain plasticity mechanisms following stroke. Results from preclinical studies and clinical observations provide evidence of an increased inhibition of neuronal function that impedes the recovery of lost neuronal function, importantly independent of the lesion size (Hagemann et al., 1998; Bütefisch et al., 2003; Xie et al., 2014). This elevated inhibitory tonus is mediated by the GABAergic system and prevents the restoration of impaired neuronal function in the area of the lesion, hindering the effectiveness of repair mechanisms such as axonal regrowth, synapse formation, and cytoskeletal rearrangement (Paik and Yang, 2014; Joy and Carmichael, 2021).

After the acute phase, layer 2/3 pyramidal neurons are exposed to a high degree of GABAergic tonic inhibition mediated by extrasynaptic receptors and attributed to GABA transporter (GAT-3/4) dysfunction (Clarkson et al., 2010). This effect lasts for up to 2 weeks after focal permanent ischemia induced by photothrombosis (Clarkson et al., 2010). Specific pharmacological inverse antagonism on the α5 subunit of GABA_A_ receptors improved functional outcomes (Clarkson et al., 2010; Wang et al., 2018). At the same time, the brain may intrinsically lower tonic inhibition. This mechanism, however, is associated with an increased frequency of epileptic discharges (Jaenisch et al., 2016).

In addition, enhanced phasic GABAergic signaling has been found in cortical layer 5 of the peri-infarct area during the first and second week of the recovery phase of stroke (Hiu et al., 2016; Feng et al., 2020). This effect seems to be mediated by α1-containing GABA_A_ receptors (Hiu et al., 2016; Neumann et al., 2019). It is of note that an increase of α1-containing GABA_A_ receptors in the synapses of lower cortical areas adjacent to the ischemic core might be highly region-specific. Interestingly, transcript levels of the α1 subunit are decreased in the proximal peri-infarct cortical area 7 days after photothrombosis (Kharlamov et al., 2008). This indicates the regulation on both the level of gene transcription as well as post-translational modifications, which may affect recycling/trafficking of receptors (Han et al., 2021). An overview on changes in the expression of GABA_A_ receptor subunits in different rodent stroke models over time is summarized in Table 1.

**Table 1:** Changes of GABA_A_ receptor subunita expression in rodent models of stroke.

Brain region	Subunits after stroke *(most common form of naive brain α1α1β2β2γ)*	Time point after stroke	Stroke model *species*	References
Infarct core	α1↓ (WB) β3↓(WB) γ2↓ (WB)	Day 2	tMCAO *mouse*	Mele et al. (2014)
Peri-infarct cortex and lateral contralateral brain	α1↓ (Immunohisto) α2↓ (Immunohisto) α3↓(Immunohisto) α5↓(Immunohisto) γ2 ↓(Immunohisto)	Day 7 Day 30	PT *rat*	Redecker et al. (2002)
Peri-infarct cortex	α1↓(Immunohisto) α2↓ (Immunohisto) α3↓(Immunohisto)	Day 7	PT *rat (young and aged)*	Schmidt et al. (2012)
Peri-infarct cortex	α5↑(Immunohisto)	Day 7	PT *rat (aged)*	Schmidt et al. (2012)
Homotopic contralateral cortex to infarct	α3↑ (Immunohisto, qPCR)	Day 7 Day 14	PT *rat and mouse*	Redecker et al. (2002) and Michalettos et al. (2021)
Motor cortex (M1) Penumbra -Layer 2/3	α4↓ (WB, qPCR) δ↓ (WB, qPCR) β3↓ (WB, qPCR)	Day 7	tMCAO *rat and mouse*	Jaenisch et al. (2016)
Peri-infarct cortex—Layer 5	α1-containing receptors↑(Array tomography)	Day 7	PT *mouse*	Hiu et al. (2016)
Peri-infarct and Contralateral cortex	α1 (RT-PCR)↓	Day 7	PT *rat*	Kharlamov et al. (2008)
Ipsilateral cortex vs. Contralateral cortex	α1 (WB)↑	Day 30	PT *rat*	Kharlamov et al. (2008)

*Abbreviations: IHC, immunohistochemistry; PT, photothrombosis; tMCAO, transient occlusion of the middle cerebral artery; WB, Western blot; qPCR, quantitative PCR. Up arrow indicates upregulation, down arrow indicates downregulation*.

Furthermore, adaptive plasticity processes in the lesioned hemisphere function together with remote neuronal networks, i.e., in the homotopic regions of non-lesioned hemisphere (Cramer, 2008; Carmichael, 2012; Boddington and Reynolds, 2017; Hakon et al., 2018). Interestingly, synaptic α3 subunits are upregulated in the contralateral motor cortex of rats subjected to photothrombosis (Redecker et al., 2002). We recently also found an interhemispheric asymmetry of the α3 subunit on transcript level during the recovery phase of stroke (Michalettos et al., 2021). This might be interpreted as intrinsic action to avert a preponderance of neuronal activity on the contralateral side. In addition, α3-containing GABA_A_ receptors may possess distinct kinetic and functional properties, regarding to their response to GABA activation (Gingrich et al., 1995; Browne et al., 2001). However, despite these studies, the exact role and regulation of synaptic GABAergic neurotransmission in the the modulation of neuronal function and plasticity remains to be studied in detail.

Data from these studies are obtained from young male rodents. Further studies will be required to understand how age and sex contribute to GABAergic neurotransmission following stroke. From a GABAergic perspective, adaptive plasticity mechanisms may be impaired in the aged brain due to a prevalenceof GABA_A_ receptors showing different pharmacological kinetics and response to GABA compared to young animals. In addition, evaluating inhibitory tonus as a measure of repressed plasticity in the post-injured brain, concomitant changes in the excitatory input may occur, making it difficult to estimate changes in the overall excitatory/inhibitory balance of the neural circuits undergoing rearrangement. In specific, both GABA_A_ and GABA_B_ receptors have been demonstrated to interact with α-amino-3-hydroxy-5-methyl-4-isoxazole propionic acid (AMPA) and N-methyl-D-aspartate (NMDA) receptors in a positive feedback manner, both on the level of neurotransmitter release and excitatory and inhibitory post-synaptic currents (Ben-Ari et al., 1997; Chen et al., 2000; Fiszman et al., 2005; Kantamneni, 2015; Schulz et al., 2018).

Despite GABA_A_ receptors being the receptor involved in the majority of cascades, activation of GABA_B_ receptors has also been proposed to participate in cellular responses mediating neuronal survival (Costa et al., 2004), further contributing to the role of GABA as an all-around protecting-mediator of neuronal injury.

### Immune/Inflammatory Response in Stroke

Inflammation is one of the core processes involved in the pathophysiology of stroke (Iadecola and Anrather, 2011). Following neuronal cell death and the initiation of the ischemic cascade, several immunological cascades take place in order to isolate and restore function to the lesioned area (Lakhan et al., 2009). However, depending on the severity of the damage and the magnitude of the immune response, secondary inflammation can further contribute to the collateral damage of the injured area, prevailing for weeks after the end of the acute ischemic phase (Rayasam et al., 2018). Processes such as microglial activation and migration, upregulation of pro-inflammatory cytokines, sealing of the injured area through glial scar formation, leucocyte chemotaxis, and infiltration, increase in blood-brain barrier (BBB) permeability, and recruitment of adaptive immunity mechanisms characterize the early environment of the brain in the acute and sub-acute phase of stroke (Morioka et al., 1993; Vila et al., 2000; Gelderblom et al., 2009; Lakhan et al., 2013; Pawluk et al., 2020). However, it is still unclear which components of the inflammatory response, either innate or adaptive, are responsible for providing a neuro-protective role and which contribute to further induced collateral damage to the lesioned ischemic tissue.

### Modulation of Glial Function by GABA—Relevance for Post-stroke Inflammation

Upon stroke, microglia, pericytes, and astrocytes, begin to seal the injured area through a process termed reactive gliosis (Burda and Sofroniew, 2014; Sims and Yew, 2017). The glial scar separates the necrotic area of the infarct core from the adjacent tissue and potentially viable neurons capable of retaining their cellular physiology. The glial scar not only provides a physical barrier but also represents a rather dynamic microenvironment regulating biochemical, intracellular, and extracellular functions in the vicinity of the injured area (Sofroniew, 2005; Becerra-Calixto and Cardona-Gómez, 2017; Sims and Yew, 2017). The physiological role of astrocytes in processes of homeostasis of neurotransmitters, transport of water, ion buffering, metabolic surveillance, and immunomodulation, has been well-established (Sofroniew, 2005; Becerra-Calixto and Cardona-Gómez, 2017; Sims and Yew, 2017). In addition, microglia, the resident immune cell of the brain, play a pivotal role in mediating inflammation and neuronal plasticity after CNS injury (Lull and Block, 2010; Anttila et al., 2017).

Interestingly, astrocytes possess components of the molecular machinery to synthesize, metabolize and store GABA, 4-aminobutyrate transaminase (GABA-T), GABA transporters as well as ionotropic and metabotropic receptors (Lee et al., 2011; Höft et al., 2014). The repertoire of GABA_A_R subunits also varies between astrocytes dependent on their localization (Riquelme et al., 2002; Höft et al., 2014). For instance, electrophysiological studies on spinal cord astrocytes showed opposite effects of inverse benzodiazepine agonism in fibrous and protoplasmic types suggesting alterations of receptor composition while undergoing morphological transitions (Rosewater and Sontheimer, 1994). In addition, astrocytes mediate neurotransmitter homeostasis through non-synaptic interactions such as uptake *via* GAT2 and GAT3 and metabolic conversion (Schousboe et al., 2013). Furthermore, with the exception of GAD, microglia also express ionotropic and metabotropic receptors as well as GABA-T (Kuhn et al., 2004; Lee et al., 2011; Nieman et al., 2020). However, the function of these receptors and transporters is poorly understood.

In contrast to astrocytes, microglia express the γ2-subunit (Höft et al., 2014; Nieman et al., 2020). It has been demonstrated that microglia actively survey and interact with synapses, extending their processes over the bulbous area of the synaptic buttons (Wake et al., 2009). The dynamics of these interactions are altered following transient middle cerebral artery occlusion (tMCAO) in mice (Wake et al., 2009). As such, microglial GABA_A_ receptors may directly associate with gephyrin complexes in the synaptic clusters, possibly affecting the trafficking of receptors (Schousboe et al., 2013). This type of direct interaction has not been verified for GABA_A_ receptors in spinal cord microglia, but rather exclusively for glycinergic receptors (Cantaut-Belarif et al., 2017). Further studies are required to evaluate similar mechanisms in brain resident microglia.

The effect of GABA on astrocytes and microglia reducing the activation of inflammatory mediators NF-κB and p38 and the release of TNFα and Il-6 after stimulation with lipopolysaccharide (LPS) and interferon-γ *in vitro* has been previously reported (Kuhn et al., 2004; Lee et al., 2011). NF-κB has been proposed as a detrimental inflammatory mediator in stroke, contributing to collateral neuronal damage (Schneider et al., 1999; Crack et al., 2006; Inta et al., 2006; Saggu et al., 2016). The Delta subunit-selective compound DS2, a positive allosteric modulator of extrasynaptic δ-containing GABA_A_ receptors (Jensen et al., 2013), has demonstrated neuroprotective properties after photothrombosis and, to some extent, the treatment exerted functional recovery following stroke by attenuation of the NF-κB response (Neumann et al., 2019). However, due to low BBB penetration, it has been speculated that the mechanism of action of DS2 is not associated with modulatory effects on brain resident glial cells but rather through functional changes in peripheral immune cells (Jin et al., 2013; Neumann et al., 2019). These findings indicate that GABA could potentially act specifically on astrocytes of the glial scar, preventing an excessive activation of NF-κB. Likewise, the reduced transcriptional activity of NF-κB in reactive astrocytes may facilitate axon regeneration and thus neural repair mechanisms (Saggu et al., 2016; Becerra-Calixto and Cardona-Gómez, 2017).

### Effects of GABA on Immune Cell Function in the Post-ischemic Brain

**Figure 1:**
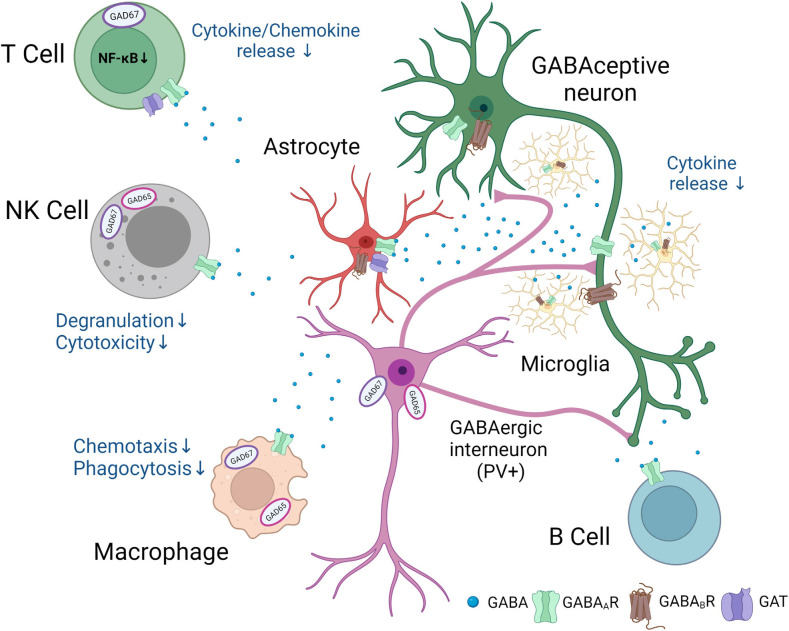
Putative involvement of GABAergic signaling in neuro-immunological crosstalk after stroke.Following stroke, neurons, glial, and immune cells synthesize and release GABA. In addition, GABA_A_ and GABA_B_ receptors and specific GABA transporters are found in a number of immune and glial cells. GABA exerts anti-inflammatory effects and changes in the function of GABAceptive neurons indicated in the figure. The figure was created with Biorender.com.

Different populations of immune cells accumulate in the ischemic territory (Gelderblom et al., 2009), integrated in an inflammatory/immune response (Iadecola et al., 2020). Functional GABA_A_ receptors have been found on microglia, dendritic cells, T cells, natural killer (NK) cells, monocytes/macrophages, B cells, and neutrophils, respectively, all cells have been reported to be involved in post-stroke inflammation. However, for most of immune cell populations, an exact link between GABA and functional changes in immune cells has not been provided following stroke. The following review of studies, therefore, summarizes potential mechanisms how GABA may regulate immune cell functions in the post-ischemic brain (Figure 1).

#### Microglia/Dendritic Cells

Microglia express both GABA_A_ and GABA_B_ receptors (Kuhn et al., 2004; Cheung et al., 2009). Activation of mainly the GABA_B_ type receptors attenuated the release of lipopolysaccharide-induced IL-6 and IL-12p40, the latter acting as a chemo attractant for macrophages and promoting the migration of dendritic cells (Cooper and Khader, 2007). In addition, GABAergic signaling has been directly linked to an increased migratory activity of dendritic cells infected with *Toxoplasma gondii* (Fuks et al., 2012).

Moreover, stimulation of microglia with either GABA or muscimol, a selective GABA_A_ receptor agonist, resulted in different levels of radical species production in cultured microglia indicating differently composed GABA_A_ receptors and intracellular cascades involved in mediating GABAergic signals (Mead et al., 2012). In addition, administration of muscimol (Lee et al., 2011) to microglia stimulated with lipopolysaccharide (LPS)/interferon-γ, the latter typically shows elevated levels following stroke (Kuric and Ruscher, 2014) significantly reduced the level of pro-inflammatory cytokines interleukin 6 (IL-6) and tumor necrosis factor alpha (TNFα). Together, results point towards anti-inflammatory actions of GABA on microglial cells. Further studies will be required to exactly determine the composition as well as functionality of different GABA_A_ and GABA_B_ type receptors in the post-ischemic brain.

#### Monocytes/Macrophages

Likewise to microglia, GABA_A_ receptors, as well as GABA synthesizing enzymes, are found in cells of the monocytic lineages (Wheeler et al., 2011). Interestingly, treatment with GABA downregulates phagocytosis and motility of macrophages and monocytic cells (Wheeler et al., 2011). Thus, an increased GABAergic tonus that develops during the first days after stroke onset might be beneficial to counteract the excessive phagocytic activity of phagocytes. In addition, GABA_A_ receptor signaling restrains “M1” activation but fosters “M2” polarization in pulmonary macrophages (Januzi et al., 2018). This is of importance since increased levels of GABA in the ischemic territory may restrain the release of cytokines that otherwise would perpetuate detrimental actions pro-inflammatory actions.

#### T Cell Populations

Similar to other immune cell populations, GABA exerts immunomodulatory actions on T cells (Bjurstöm et al., 2008; Dionisio et al., 2011). Effects of an increased GABAergic tonus on the number of different T cells subpopulations after stroke have not been determined. Hence, GABA contributes to TCR-mediated T cell cycle progression silencing CD4^+^ T cells in the G0/G1 phase consisting of a higher portion of CD3+/CD28+ cells without affecting their viability (Tian et al., 2004). In addition, it has been demonstrated that administration of GABA or homotaurine, a GABA_A_ receptor-specific agonist, is involved in increasing the number of regulatory T cells in EAE as well as type1 Diabetes models (Tian et al., 2018). Specifically, in the experimental autoimmune encephalitis (EAE) model, treatment enhanced the number of CD8+CD122+PD-1+ and CD4+Foxp3+ Treg cells. Regulatory CD19+IL-10+ B cells were not affected. Interestingly, IL-10+B-cell treated mice show an increased number of IL-10+CD8+CD122+ Treg population. Generation of these cells has been associated with spleen preservation and reduced CNS inflammation after tMCAO (Bodhankar et al., 2015). The role of CD4+Foxp3+ Treg cells in stroke recovery, however, remains divergent in might be dependent on the stroke model, different microenvironments in the post-ischemic brain as well as the time intervals after the insult (Liesz and Kleinschnitz, 2016). In addition, homotaurine inhibits autoreactive Th17 and Th1 responses as well as relevance for stroke recovery processes. Stimulation of T cells with GABA also has been shown to inhibit the production of pro-inflammatory cytokines and therefore it attenuates the T cell response in inflammatory disease models such as EAE but also in Diabetes models (Soltani et al., 2011; Prud’homme et al., 2015). In addition to GABA_A_ receptor activation-mediated effects on T cells, a reasonable number of publications demonstrate some of the GABAergic effects are at least partially mediated by activating the GABA transporter type 1 (GAT-1; Wang et al., 2008). It is exclusively expressed on activated T cells primed with antigens. Increased influx of GABA *via* the transporter downregulates proliferation of the CD4+ T cells (Wang et al., 2008).

#### Natural Killer Cells

NK cells represent a population of innate immune cells accounting for about 5%–20% of human blood (Perera Molligoda Arachchige, 2021). Recently, this population has been defined as GABAergic cells due to the release of the neurotransmitter upon stimulation/exposure to pathogens and/or inflammatory stimuli (Bhandage et al., 2021). This would predestine NK cells’ interaction with other immune cells and brain resident GABAceptive cells. One possible GABA-driven interaction comes from experiments performed in NK cells and dendritic cells infected with *Toxoplasma gondii* (Bhandage et al., 2021). Here, stimulation with exogenous GABA reduced degranulation and cytotoxicity of NK cells. Conversely, NK cells conditioned medium containing GABA enhanced migration of parasitized dendritic cells. Such interactions might be of relevance in the post-ischemic brain since both cell types significantly contribute to post-stroke inflammatory cascades (Gan et al., 2014).

### Impact of Chemokine Pathways on GABAergic Neurotransmission

Chemokines and respective receptor driven inflammatory cascades have been identified as an essential component in stroke recovery mechanism and may offer a promising field identifying novel targets to improve functional outcome.

The absence of the C-C chemokine receptor type 5 (CCR5) receptor, for example, results in worse outcomes in mice after stroke exhibiting bigger infarct sizes, sustained invasion of neutrophils during the first 7 days, and reduced brain plasticity in the chronic recovery phase (Sorce et al., 2010; Ping et al., 2021). In contrast, conditional knockdown of neuronal CCR5 prior to stroke or pharmacological antagonism of CCR5 1 day after the stroke incident were shown to be beneficial for the neurological outcome and enhanced brain plasticity (Joy et al., 2019). Similarly, the C-X3-C Motif Chemokine Ligand 1/C-X3-C Motif Chemokine Receptor 1 (CX3CL1/CX3CR1) pathway, which is unique in the CNS, has been implied to possess divergent functions. While CX3CL1 or CX3CR1 deficiency points towards a neuroprotective role (Soriano et al., 2002; Dénes et al., 2008), administration of CXC3L1 to wild-type mice or cx3cl1−/− mice showed reduced ischemic lesions in wildtype animals while an increase in lesion sizes was found in knockout littermates, respectively (Cipriani et al., 2011). We have previously reported that CX3CR1 deficiency does not affect infarct size and outcome, but causes alterations in the morphology of microglia populating the peri-infarct area (van der Maten et al., 2017).

Pharmacological antagonism of C-X-C Motif Chemokine Receptor 1/C-X-C Motif Chemokine Receptor 2 (CXCR1/CXCR2) by the C-X-C Motif Chemokine Ligand 8 (CXCL8) receptor blocker reparixin aiming at attenuating leukocyte infiltration promoted functional outcome and reduced infarct size (Villa et al., 2007). Likewise, C-C Motif Chemokine Receptor 2CCR2−/− knockout mice exhibited beneficial outcome after tMCAO, with reduced monocyte and macrophage infiltration as well as reduced BBB permeability (Dimitrijevic et al., 2007). We have previously shown that pharmacological antagonism of C-X-C Motif Chemokine Receptor 4 (CXCR4) with AMD3100 attenuates the accumulation of CX3CR1-positive microglia and contributes to enhanced recovery of lost neurological function (Walter et al., 2015). Likewise, conditional knockout of the CXCR4 gene in hematopoietic stem cells (HSCs) and their derivatives, such as circulating monocytes and monocyte-derived macrophages, results in a reduced population of immune cells in the ischemic territory, after both PT and tMCAO (Werner et al., 2020).

Besides their function on inflammatory cells, experimental evidence emerge revealing a role of chemokine-driven cascades in neurons and neuronal function in the post-ischemic brain (de Haas et al., 2007). Following a stroke, an upregulation of CCR5 transcripts has been detected in neuronal cells, which was not detectable before the insult and in naïve mice (Joy et al., 2019). To which extent transcripts are translated into functional proteins remains to be elucidated. We previously found that NeuN+ neurons express CXCR4 in the peri-infarct area, as a target for AMD3100 treatment to specifically modulate this pathway (Ruscher et al., 2013). Similarly, CCL2 and CX3CR1 are upregulated in neurons following hypoxia or ischemia (Andres et al., 2011; Wang et al., 2018). CCR2 has also been demonstrated to affect GABA-induced currents in spinal neurons, indicating mechanistic interactions between chemokine receptors and GABA_A_ receptors (Gosselin et al., 2005). GABA-induced currents are also affected by cytokines (Giacco et al., 2019). Susceptibility of neurons to chemokines and cytokines potentially modulates mechanisms of synaptic plasticity, neurotransmitter receptor expression and neurotransmitter-producing enzymes. Therefore, we will summarize the current evidence on how chemokine pathways interact with the GABAergic neurotransmission following stroke.

### Cross Talk Between the CX3CL1/CX3CR1 Pathway and GABAergic Neurotransmission

It has been previously described that CX3CL1, as a membrane-bound protein found both in neurons and glial cells, undergoes cleavage after excitotoxic conditioning. Shedding of the protein is characteristic of the ischemia onset (Chapman et al., 2000; Meucci et al., 2000; Wang et al., 2018). As such, the soluble form of the chemokine acts in a paracrine fashion on microglia and neurons, as well as a chemotactic agent for infiltrating immune cells (Imai et al., 1997; Dichmann et al., 2001; Tarozzo et al., 2002; Wang et al., 2018). Electrophysiological studies on hippocampal neurons and serotonin neurons of the dorsal raphe nucleus have demonstrated that the application of CX3CL1 enhances inhibitory post-synaptic currents through GABA_A_ receptors but depresses excitatory inputs from AMPA receptors through post-translational modifications (Ragozzino et al., 2006; Heinisch and Kirby, 2009). From a GABAergic perspective, the neuroprotective role of CX3CL1 could be attributed in part to enhanced inhibitory currents and suppressed AMPA receptor function during the acute excitotoxic phase of ischemia (Cipriani et al., 2011).

Studies on both physiological and pathological conditions, such as epilepsy, demonstrate that the CX3CR1 pathway may regulate the number of post-synaptic GABA_A_ receptors or their sensitivity to GABA and thus their subunit composition (Heinisch and Kirby, 2009; Roseti et al., 2013). We have shown that CX3CR1 deficiency modulates the expression of GABA_A_ receptor subunits in the recovery phase of stroke both in the ischemic and contralateral to the lesion hemisphere (Michalettos et al., 2021). This indicates that plastic procedures occurring in the homotopic contralateral motor region may be partially mediated by a CX3CR1-dependent mechanism. However, it needs to be further elucidated whether this effect is the result of neuronal receptor absence or an altered microglia-synapse interaction (Wake et al., 2009).

### Involvement of CXCR4/CXCL12 in Neuromodulation After Stroke

The upregulation of the CXCR4/CXCL12 pathway in the ischemic hemisphere has been extensively described (Stumm et al., 2002; Schönemeier et al., 2008; Wang et al., 2012; Ruscher et al., 2013). However, there is limited data on the aspect of how the CXCR4/CXCR7/CXCL12 axis modulates neuronal function following stroke. It is well established that post-natal neurons express functional CXCR4 receptors, including GABAergic interneurons (Trecki et al., 2010; Wu et al., 2017). Electrophysiological studies point towards a pre-synaptic mode of action of the receptor, mediating Ca^2+^-dependent release of GABA (Guyon et al., 2006; Heinisch and Kirby, 2010), and a post-synaptic mode of action, possibly involving direct interactions of the receptor with GABA_B_ receptors (Guyon et al., 2006). In addition, it has been demonstrated that CXCL12 modulates CX3CL1 homeostasis by regulating CX3CL1 expression as well as CX3CL1 cleavage rate under physiological conditions (Cook et al., 2010). We have confirmed this interaction *in vivo*, in the recovery phase of mice subjected to PT, in which pharmacological antagonism with AMD3100 resulted in reduced CX3CL1 levels, both membrane-bound and soluble (Walter et al., 2015). Therefore, low levels of CX3CL1 may shift the inhibitory-excitatory balance towards an excitatory tissue environment, allowing for beneficial plastic procedures to take place, a process which might be age-dependent.

As such, the potential effects of AMD3100 on the inhibitory-excitatory balance of the post-ischemic brain involve two distinct levels of interactions, including modulation of inflammatory response and regulation of neuronal function. Increased levels of GAD enzymes and GAD67-positive neurons have been observed in the ipsilateral striatum 1 to 2 days after transient forebrain ischemia (Li et al., 2010). An increase of inhibitory neurons in the vicinity of the ischemic tissue was attributed to resident somatostatin-expressing neurons shifting to a GABAergic phenotype and not the maturation of migrating neural precursor cells (Li et al., 2010). *In vitro* studies, on the other hand, showed that the CXCR4 pathway is related to the maturation process of embryonic hippocampal neurons through the induction of GAD67 expression (Luo et al., 2008). We have demonstrated that administration of AMD3100 for 2 weeks resulted in decreased expression of GAD67 and GABA_A_ subunits in the peri-infarct area (Michalettos et al., 2021). This might be related to a reduced formation of GAD67-positive interneurons or direct regulation of GAD67 expression downstream of neuronal CXCR4 receptors. The downregulation of GABA_A_ receptors by the treatment was not subunit-specific. Therefore, it is likely that inflammatory mediators are responsible for the synthesis/turnover of several types of GABA_A_ receptors. Further research is required to delineate the exact mechanism of action of CXCR4 regarding the regulation of GABA_A_ receptors in healthy and injured neurons undergoing adaptive plastic procedures.

## Conclusions

Based on solid preclinical studies, post-stroke GABAergic neurotransmission and detrimental inflammatory cascades have been targeted in clinical trials. However, studies did not meet primary endpoints for several reasons. From the preclinical point of view, we are beginning to understand the complex interaction between inflammatory cascades and neuronal functions. Only with full comprehension about pathophysiology of adaptive neuronal plasticity and definitions in conjunction with well-designed clinical trials will allow for implementation of new adjuvant treatments to enhance neurological functions after ischemic stroke.

## Author Contributions

Both authors contributed equally to the first draft and revision of the mansucript. All authors contributed to the article and approved the submitted version.

## Conflict of Interest

The authors declare that the research was conducted in the absence of any commercial or financial relationships that could be construed as a potential conflict of interest.

## Publisher’s Note

All claims expressed in this article are solely those of the authors and do not necessarily represent those of their affiliated organizations, or those of the publisher, the editors and the reviewers. Any product that may be evaluated in this article, or claim that may be made by its manufacturer, is not guaranteed or endorsed by the publisher.

## Abbreviations

AD: Alzheimer’s disease
AMD3100: 1,1^′^-[1,4-phenylenebis(methylene)]bis[1,4,8,11-tetraazacyclotetradecane]
CCR2: C-C chemokine receptor type 2
CCR5: C-C chemokine receptor type 5
CX3CL1: C-X3-C Motif Chemokine Ligand 1
CX3CR1: C-X3-C Motif Chemokine Receptor 1
CXCL8: C-X-C Motif Chemokine Ligand 8
CXCR4: C-X-C Motif Chemokine Receptor 4
CNS: central nervous system
EAE: experimental autoimmune encephalitis
GABA: γ-aminobutyric acid
GABA_A_R: γ-aminobutyric acid type A receptor
GABA_B_R: γ-aminobutyric acid type B receptor
GAD65: glutamic acid decarboxylase 65
GAD67: glutamic acid decarboxylase 67
GAT: γ-aminobutyric acid transporter
GIRK: G protein-gated inward rectifying potassium
NK: natural killer
NF-κB: nuclear factor kappa-light-chain-enhancer of activated B cells
PT: photothrombosis
TCR: T-cell receptor
tMCAO: transient middle cerebral artery occlusion
TNFα: Tumor necrosis factor alpha
VSCC: voltage-sensitive calcium channel.
